# Promoting well-being: Investigating self-efficacy and academic burnout among trainee teachers

**DOI:** 10.1192/j.eurpsy.2024.1653

**Published:** 2024-08-27

**Authors:** Z. Boumaaize, A. Bouhaba, M. A. Lafraxo, H. Guider, Y. El Madhi, H. Hami

**Affiliations:** ^1^Laboratory of Biology and Health, Faculty of Sciences, Ibn Tofail University, Kenitra; ^2^Regional Center for Education and Training Professions, Rabat, Morocco

## Abstract

**Introduction:**

Burnout is a widespread problem with far-reaching implications for mental health. Recent studies on working conditions in Morocco have drawn attention to the increasing prevalence of psychosocial hazards, notably stress and burnout, in various professions. The emergence of burnout is mainly determined by the intricate interplay of organizational, environmental, and individual factors. In education, the teaching profession is susceptible to various burnout symptoms. Educators can mitigate this syndrome by maintaining a positive outlook driven by a strong sense of self-efficacy.

**Objectives:**

This study investigated the correlation between academic burnout syndrome and personal resources, specifically a sense of efficacy.

**Methods:**

A cross-sectional survey of 160 Moroccan trainee teachers, with an average age of 27.94±5.44 years, was conducted. Data were collected through a self-administered questionnaire that included the Maslach Burnout Inventory-Student Survey (MBI-SS) and Teachers’ Sense of Efficacy Scale (TSES). The MBI-SS evaluated academic burnout across three dimensions: emotional exhaustion, cynicism, and academic efficacy, whereas the TSES examined efficacy for classroom management, student engagement, and instructional strategies. The questionnaires were translated into Arabic and validated for use in the Moroccan context.

**Results:**

The findings revealed a moderate and statistically significant correlation between efficacy for classroom management and the two components of efficacy related to instructional strategies (r=0.32; p<0.001) and student engagement (r=0.49; p<0.001). Additionally, a significant and positive correlation was observed between instructional strategies’ efficacy and the efficacy for student engagement (r=0.23; p<0.01). A moderate and significant correlation was found between emotional exhaustion and cynicism (r=0.45; p<0.001), whereas academic efficacy and cynicism were negatively and significantly correlated (r=-0.13; p<0.05). It is worth noting that the key component of academic burnout, “emotional exhaustion,” was significantly related to academic efficacy (r=-0.58; p<0.001). Additionally, Pearson’s correlation test demonstrated a positive and statistically significant correlation between emotional exhaustion and efficacy for student engagement (r=0.14; p<0.05). Furthermore, the correlation between academic burnout and self-efficacy showed a negative and statistically significant association (r=-0.13; p<0.05).

**Conclusions:**

Trainee teachers face a range of stressors that affect their well-being. By focusing on personal traits, well-being can be improved and burnout mitigated. This study highlights the key role of self-efficacy as a critical resource in preventing academic burnout, particularly among teachers at the start of their careers.

**Disclosure of Interest:**

None Declared

